# Machine learning for the classification of serial electron diffraction patterns: synthetic data

**DOI:** 10.1107/S2053273325005327

**Published:** 2025-07-07

**Authors:** Tatiana E. Gorelik, Evgeny Gorelik

**Affiliations:** a Ernst Ruska-Centre for Microscopy and Spectroscopy with Electrons Forschungszentrum Jülich Jülich 52428 Germany; b Technische Universität Berlin Straße des 17. Juni, 135 Berlin 10623 Germany; University of Warsaw, Poland

**Keywords:** serial electron crystallography, machine learning

## Abstract

Serial electron crystallography faces a fundamental challenge due to the flat Ewald sphere resulting from the short electron wavelength, leading to limited 3D information in individual patterns. Recently, an algorithm for unit-cell determination from zonal electron diffraction patterns (GM algorithm) [Miehe (1997). *Ber. Dtsch. Miner. Ges. Beih. z. Eur. J. Miner.***9**, 250; Gorelik *et al.* (2025). *Acta Cryst.* A**81**, 124–136] was introduced in the context of serial electron crystallography. This algorithm requires the extraction of 2D zonal patterns from the complete serial dataset. Here, we present a machine learning approach for pattern sorting and apply it initially to simulated electron diffraction patterns.

## Abstract 2

Machine learning based sorting of synthetic serial electron diffraction patterns into 2D zonal patterns and patterns representing intersections of multiple Laue zones is demonstrated. The extracted zonal patterns can be used for the determination of unit-cell parameters.

Crystal structure determination becomes challenging when the crystal size is smaller than a micrometre. To address this challenge, serial crystallography was developed (Chapman *et al.*, 2011 ▸), a technique in which thousands or even millions of diffraction snapshots are collected from a large number of individual crystals. Bragg intensity measurements from each diffraction pattern are then combined into a single merged dataset, which is used for structure determination.

Serial crystallography was originally developed for X-ray free-electron lasers (XFELs), where each X-ray pulse destroys the crystal. It has since been adapted for synchrotron light sources, where it is particularly useful for time-resolved experiments. In the meantime, serial crystallography is primarily used for protein structure determination (Spence, 2017 ▸).

Recently, a small-molecule crystal structure was determined using serial XFEL crystallography (Takaba *et al.*, 2023 ▸). The small unit-cell parameters of the compound resulted in a lower density of reflections in reciprocal space compared with proteins, leading to fewer diffraction spots per frame. As a result, the unit-cell parameters could not be determined directly from the data and were instead supplied from a complementary 3D electron diffraction (3D ED) (Gemmi *et al.*, 2019 ▸) measurement (Takaba *et al.*, 2023 ▸).

A serial crystallography experiment produces a massive amount of data, with only a small fraction containing diffraction patterns from crystals that can be used for crystallographic analysis. In this context, the primary task of data processing is to sort the patterns into *no-hit* (to be discarded) and *hit* patterns (to be further processed). This categorization task can be performed either by statistical methods – by detecting Bragg peaks in a pattern and discarding those with fewer than a threshold number of peaks – or, increasingly, by machine learning techniques, based on different architectures, aiming to recognize entire patterns as *hits* or *no-hits* in a manner similar to human visual perception (Ke *et al.*, 2018 ▸; Nawaz *et al.*, 2023 ▸; Rahmani *et al.*, 2023 ▸; Rahmani *et al.*, 2024 ▸). Meanwhile, large labelled experimental and synthetic datasets (Souza *et al.*, 2019 ▸) have become available for training, facilitating further developments in this direction.

The feasibility of serial electron crystallography has already been demonstrated in a series of studies (Smeets *et al.*, 2018 ▸; Bücker *et al.*, 2020 ▸; Plana-Ruiz *et al.*, 2023 ▸; Hogan-Lamarre *et al.*, 2024 ▸). Serial electron crystallography offers several advantages over synchrotron-based methods. Compared with large synchrotron facilities, transmission electron microscopes are more accessible and cost-effective. The technique also provides greater flexibility in experimental geometry, as data can be collected in either transmission electron microscopy (TEM) or scanning TEM (STEM) mode, enabling targeted crystal selection and significantly reducing the number of *no-hit* patterns. In practice, this makes the pre-categorization of electron diffraction patterns into *hits* and *no-hits* nearly obsolete. Additionally, sample preparation and handling are considerably easier.

However, these benefits come with a particular challenge: the much shorter wavelength of electrons compared with X-rays results in an almost flat Ewald sphere. As a consequence, diffraction patterns appear nearly flat, making unit-cell determination from serial electron data even more difficult. All studies on serial electron crystallography reported so far have used unit-cell parameters obtained from other sources – either the unit cell was already known, or it was determined using a complementary technique such as 3D ED.

Electron diffraction patterns recorded in a serial experiment can be classified into two groups: *zonal patterns* and *3D patterns*, which represent sections through multiple Laue zones. Zonal patterns contain only a 2D net of reflections and were extensively used in the early days of electron crystallography *e.g.* to determine the symmetry of materials (Steeds & Vincent, 1983 ▸). By definition, these patterns do not contain any 3D information. In contrast, randomly oriented 3D sections of reciprocal space do carry 3D information, and these patterns gained prominence with the development of the 3D ED method (Gemmi *et al.*, 2019 ▸).

Recently, the GM algorithm – originally developed in the 1990s for unit-cell determination from zonal ED data – was introduced (Miehe, 1997 ▸; Gorelik *et al.*, 2025 ▸). Interestingly, a similar algorithm was developed by Belletti *et al.* (2000 ▸), suggesting that this topic was actively explored decades ago, well before the advent of serial crystallography.

The GM algorithm has the potential to enable fully *ab initio* structure determination for serial electron diffraction data without requiring complementary information on unit-cell parameters. It takes a few zonal patterns as input, making the automatic extraction of zonal patterns from a full serial electron crystallography dataset (potentially containing thousands of patterns) a crucial task.

To address this challenge, we explored the use of machine learning classification techniques. The fundamental differences in the geometry of electron and X-ray diffraction patterns imply that existing labelled training data for X-rays cannot be directly applied to electron diffraction. Therefore, we simulated randomly oriented electron diffraction patterns to mimic the outcome of a serial electron diffraction experiment.

Electron diffraction (ED) patterns were simulated using the following parameters:

(i) The unit-cell volume, which determines the reflection density in reciprocal space. A random unit cell was generated with lattice parameters within a specified range. We varied these ranges to produce both nearly isotropic unit cells (with similar unit-cell lengths) and strongly anisotropic unit cells, ensuring coverage of all possible cases. Initially, we focused only on triclinic cells with the unit-cell volume of 500, 700 and 1000 Å^3^.

(ii) An electron wavelength of 0.0251 Å, corresponding to 200 kV electrons.

(iii) ED data resolution of 1 Å^−1^.

(iv) An excitation error of 0.01 Å^−1^.

For a given set of unit-cell parameters, 1000 patterns were calculated, each representing a different lattice orientation. These orientations were generated using the Fibonacci sphere method. The patterns were produced as black-and-white images, with a slightly enlarged central spot in the middle of the pattern (Fig. 1 ▸) representing the primary beam. All reflections had uniform size and intensity.

For training data, we manually labelled the patterns, classifying them into 2DZone and 3DLaueIntersections categories. Fig. 1 ▸ shows typical representatives of these classes. In total, 4000 patterns were used for training.

While labelling the patterns into 2DZone and 3DLaueIntersections classes, we observed that the fraction of zonal patterns was very low. For a unit-cell volume of 1000 Å^3^, an electron wavelength of 0.0251 Å, a data resolution of 1 Å and an excitation error of 0.01 Å−^1^, only 3% of the patterns fell into the 2DZone category. It is important to note that we applied a very strict criterion for the 2DZone class – the pattern had to contain only reflections forming a visible 2D net, with no additional reflections beyond it.

For training, we used the Faster R-CNN architecture (Ren *et al.*, 2015 ▸) with a ResNet-50 backbone (He *et al.*, 2016 ▸) pre-trained on the COCO dataset (Lin *et al.*, 2014 ▸). Although newer architectures, such as transformer-based models (Dosovitskiy *et al.*, 2021 ▸), are available and can outperform our chosen network, the Faster R-CNN model was sufficient for differentiating between two classes in our synthetic binary data. Moreover, it was readily available in the *PyTorch* model zoo and required minimal modification for adaptation to our experiment.

Training was conducted using the *PyTorch* framework with GPU acceleration on an RTX 2080, over 10 epochs, selecting the best-performing model based on validation set performance. Images were downscaled to 254 × 254 pixels, and the number of output classes was set to 2 (2DZone and 3DLaueIntersections), replacing the 91 classes of the original COCO dataset. All labelled data were split into training and validation sets in an 80:20 ratio.

As the neural network was pre-trained on the COCO dataset, thereby being generalizable to many use-cases, fine-tuning the neural network on the task of 2DZone detection required little training time. Running the training pipeline for a dataset containing approximately 1000 samples for 7 epochs took around 10 min on an RTX 2080 GPU. Inference on the same device has a throughput rate of approximately 10 samples per second.

One issue encountered in the initial training iterations was a significant class imbalance. Since the number of zonal samples was much smaller than that of 3DLaueIntersections samples, the neural network achieved high overall accuracy by classifying all samples as 3DLaueIntersections, effectively neglecting the misclassification of zonal samples. To address this imbalance, we implemented a class-balanced sampling strategy for training, where the probability of selecting a sample was inversely proportional to the class size. This approach significantly improved prediction performance, ultimately achieving 100% accuracy on our validation dataset.

The result of the sorting process was the assignment of each pattern to one of the two classes, along with a confidence level. A confidence of 1 generally indicated a clear case of a 2DZone or 3DLaueIntersections pattern.

Interesting cases arose when the confidence level was lower than 1. In most instances, these patterns were close to a 2D zone but contained a few additional reflections from a different Laue layer, exhibiting a hybrid behaviour (Fig. 2 ▸). In principle, such patterns could still be used for the GM algorithm by extracting vectors from the dominant 2D pattern.

We trained the model on four triclinic unit cells with a volume of 1000 Å^3^, then tested its performance on smaller unit cells with volumes of 500 and 700 Å^3^, achieving very good results. Finally, we generated unit cells with a monoclinic metric (*a* = 3.8155, *b* = 10.0452, *c* = 13.7425 Å, β = 107.8717°; *a* = 3.7061, *b* = 12.0249, *c* = 11.7474 Å, β = 103.1097°) and introduced extinctions along the *b* axis, corresponding to the effect of a 2_1_ screw axis. Despite not being trained on data with extinctions, the model successfully classified the patterns correctly.

The success of the classification procedure was remarkable. This may be because we used simulated data, without the uncertainties inherent in experimental ED patterns, such as detector noise and gain, potential data pathologies, including *no-hit* patterns, multiple crystals in a pattern, crystal mosaicity and errors in peak position determination. Nevertheless, we demonstrated that the proposed architecture effectively sorts patterns into two distinct classes. This approach can be easily expanded to include additional categories, such as *no-hit* patterns and multiple overlying crystals, which can be handled separately in subsequent serial data processing steps.

In summary, we present a machine learning approach to classify simulated ED patterns into two categories: 2DZone or 3DLaueIntersections. Patterns assigned to the 2DZone class can subsequently be used for unit-cell determination using the GM algorithm (Miehe, 1997 ▸; Gorelik *et al.*, 2025 ▸). While machine learning techniques have already been applied in X-ray crystallography to categorize patterns into hits and no-hits (Ke *et al.*, 2018 ▸; Souza *et al.*, 2019 ▸; Nawaz *et al.*, 2023 ▸; Rahmani *et al.*, 2023 ▸; Rahmani *et al.*, 2024 ▸), we take a step further by analysing the internal structure and features of the patterns themselves.

In general, two strategies can be pursued in the future for sorting experimental ED patterns: (i) training models directly on experimental data, or (ii) extracting peak positions from experimental patterns – an inherent step in serial crystallography data processing – and then generating semi-synthetic patterns based on these peaks for classification. The latter approach could help decouple the influence of the detector from the data during training, although in practice it shifts the consideration of detector characteristics to the peak extraction step. Future investigations will determine which approach proves more robust, efficient, and practical to implement.

## Figures and Tables

**Figure 1:**
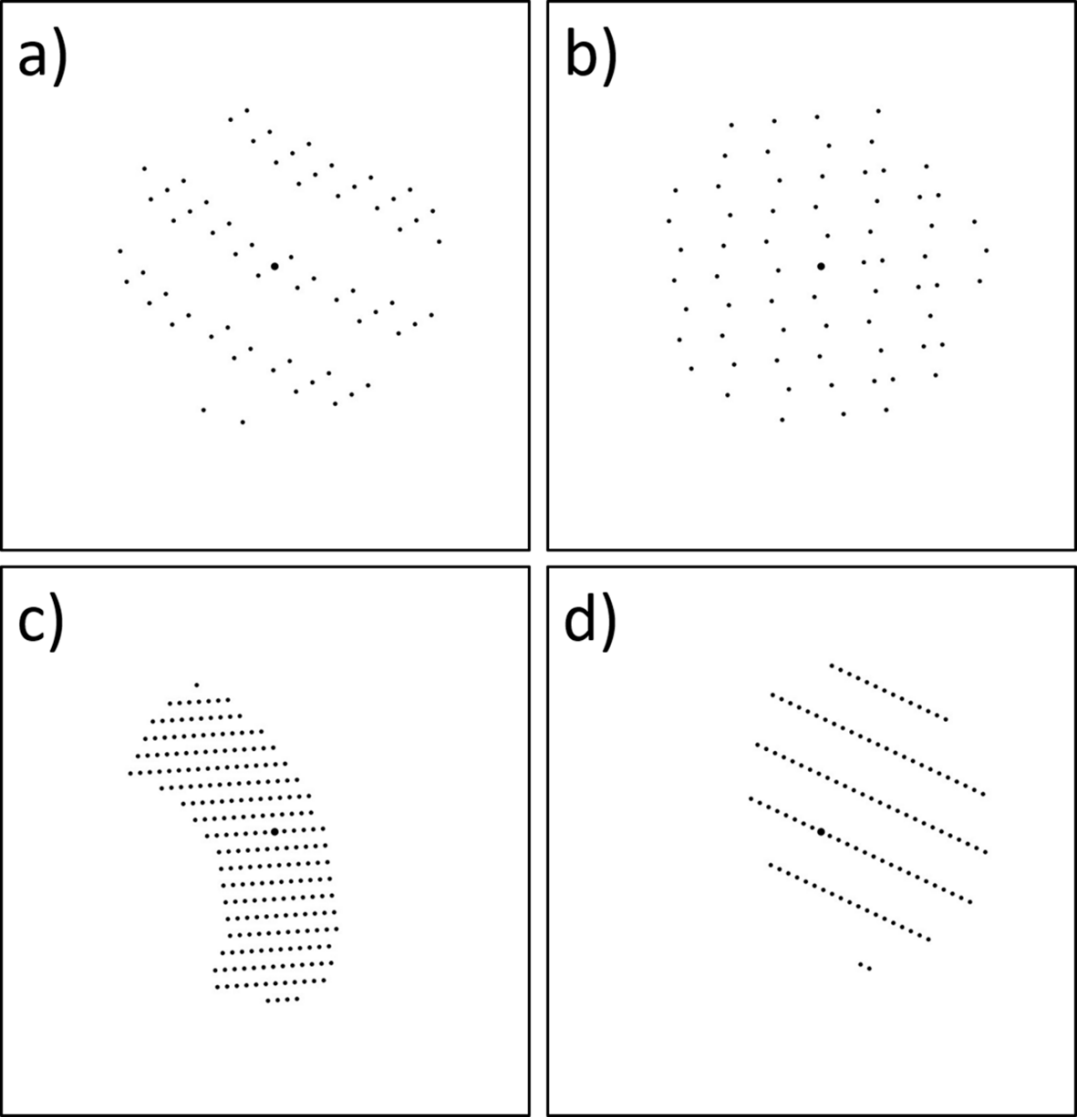
Typical patterns from the training data: (*a*) and (*b*) 3DLaueIntersections, (*c*) and (*d*) 2DZone. Unit-cell parameters: *a* = 5.6786, *b* = 10.4648, *c* = 20.4682 Å, α = 109.7914°, β = 106.5805°, γ = 105.8206°.

**Figure 2:**
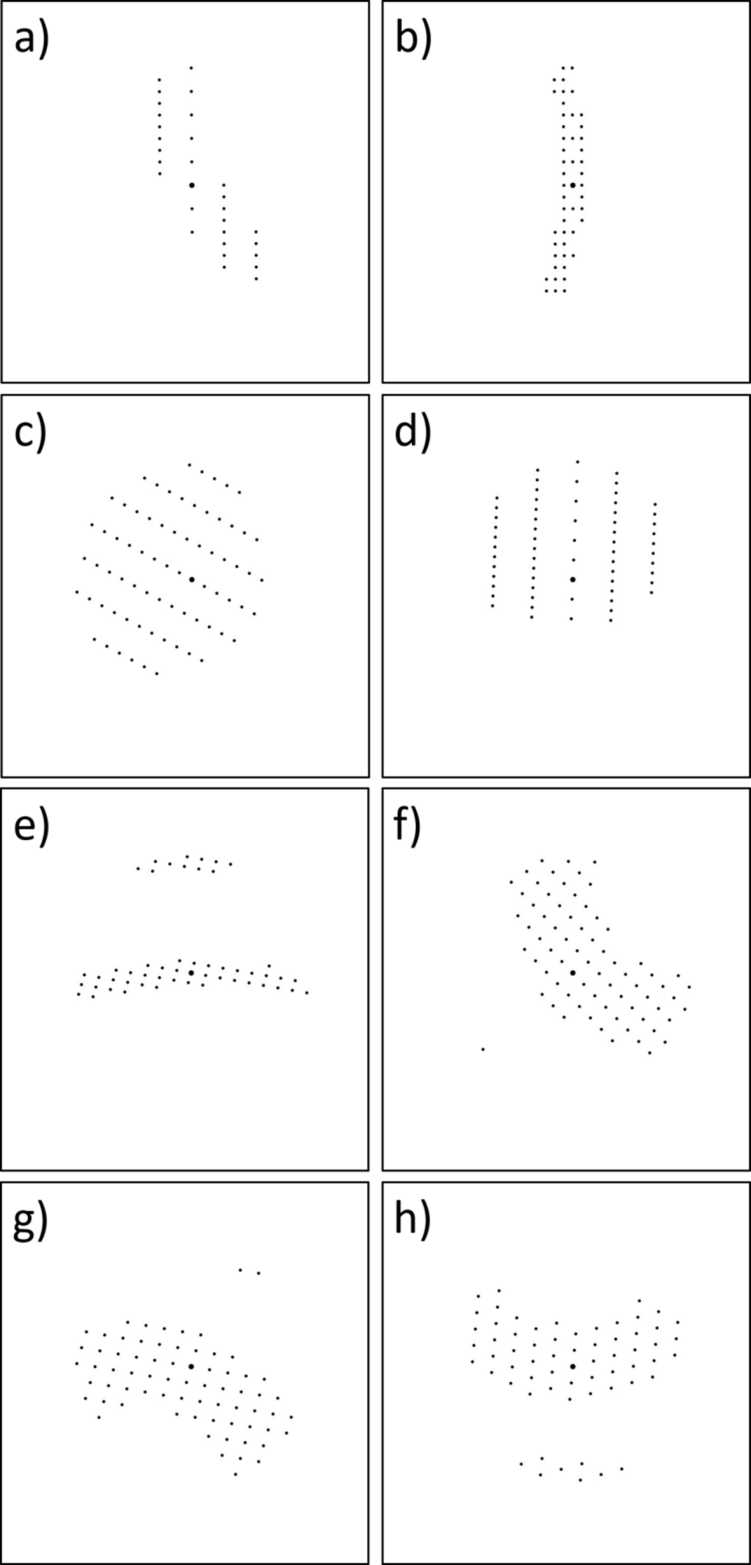
Members of the 2DZone class with confidence values less than 1. Monoclinic unit cell with cell parameters *a* = 3.8155, *b* = 10.0452, *c* = 13.7425 Å, β = 107.8717°: (*a*) confidence 0.9906, (*b*) 0.9999. Monoclinic unit cell with cell parameters *a* = 3.7061, *b* = 12.0249, *c* = 11.7474 Å, β = 103.1097°: (*c*) confidence 0.8651, (*d*) 0.8948. Triclinic unit cell with cell parameters *a* = 9.7733, *b* = 11.979, *c* = 9.6421 Å, α = 91.1645°, β = 113.204°, γ = 102.2199°: (*e*) confidence 0.7382, (*f*) 0.9408. Triclinic unit cell with cell parameters *a* = 6.4633, *b* = 9.4541, *c* = 19.4332 Å, α = 104.2276°, β = 118.6144°, γ = 90.5482°: (*g*) confidence 0.7575, (*h*) 0.6471.

## Data Availability

The code for training and running inference on the model is available at: https://github.com/EvgenyGorelik/geometric-diffraction-analysis. Simulated electron diffraction patterns used in this study (training data and sorted patterns) as well as the relevant *MATLAB* codes for data generation and sorting are available at: https://doi.org/10.5281/zenodo.14925441.
